# Genomic architecture of sickle cell disease in West African children

**DOI:** 10.3389/fgene.2014.00026

**Published:** 2014-02-14

**Authors:** Jacklyn Quinlan, Youssef Idaghdour, Jean-Philippe Goulet, Elias Gbeha, Thibault de Malliard, Vanessa Bruat, Jean-Christophe Grenier, Selma Gomez, Ambaliou Sanni, Mohamed C. Rahimy, Philip Awadalla

**Affiliations:** ^1^Department of Social and Preventive Medicine, Faculty of Medicine, School of Public Health, University of MontrealMontreal, QC, Canada; ^2^Department of Pediatrics, Faculty of Medicine, Sainte-Justine Research Center, University of MontrealMontreal, QC, Canada; ^3^Biology, Division of Science and Mathematics, New York University Abu DhabiAbu Dhabi, United Arab Emirates; ^4^Laboratoire de Biochimie et Biologie Moléculaire, Faculté des Sciences et Techniques, Université d'Abomey-CalaviCotonou, Benin; ^5^Faculté des Sciences de la Santé, Centre de Prise en charge Médicale Intégrée du Nourrisson et de la Femme Enceinte atteints de Drépanocytose, Université d'Abomey-CalaviCotonou, Benin

**Keywords:** sickle cell disease, genomics, transcriptome, eSNP mapping, gene-by-environment interactions

## Abstract

Sickle cell disease (SCD) is a congenital blood disease, affecting predominantly children from sub-Saharan Africa, but also populations world-wide. Although the causal mutation of SCD is known, the sources of clinical variability of SCD remain poorly understood, with only a few highly heritable traits associated with SCD having been identified. Phenotypic heterogeneity in the clinical expression of SCD is problematic for follow-up (FU), management, and treatment of patients. Here we used the joint analysis of gene expression and whole genome genotyping data to identify the genetic regulatory effects contributing to gene expression variation among groups of patients exhibiting clinical variability, as well as unaffected siblings, in Benin, West Africa. We characterized and replicated patterns of whole blood gene expression variation within and between SCD patients at entry to clinic, as well as in follow-up programs. We present a global map of genes involved in the disease through analysis of whole blood sampled from the cohort. Genome-wide association mapping of gene expression revealed 390 peak genome-wide significant expression SNPs (eSNPs) and 6 significant eSNP-by-clinical status interaction effects. The strong modulation of the transcriptome implicates pathways affecting core circulating cell functions and shows how genotypic regulatory variation likely contributes to the clinical variation observed in SCD.

## Introduction

Sickle cell disease (SCD) is an autosomal recessive genetic disorder particularly common among individuals of Sub-Saharan African ancestry, affecting 1 in 100 West African individuals and 1 in 500 African-Americans (World Health Organization, 2006). Genetic mutations that cause SCD result in structural changes to wild-type hemoglobin (HbAA), the oxygen carrying protein inside red blood cells (RBCs). The most common form of SCD in West Africa is caused by a single point mutation in codon 6 of the β-globin gene which leads to an amino acid substitution of glutamic acid to valine (HbSS). The second most common abnormal Hb mutation in West Africa, HbC, results in an amino acid change at the same position in the beta globin gene, but with lysine replacing glutamic acid. These hemoglobin mutations compromise the delivery of oxygen and result in tissue and organ damage. Despite the monogenic origin of the disease, SCD patients exhibit a broad spectrum of clinical variation (Driss et al., 2009) ranging from patients with mild forms of the disease that rarely require medical interventions to patients with severe complications warranting frequent hospitalization and aggressive clinical follow-up. Homozygous HbSS and compound heterozygous HbSC individuals suffer from SCD with overlapping yet distinctive clinical and biochemical features (Hannemann et al., 2011). Inter-individual clinical variation is also pervasive within each of these SCD groups, but its basis is poorly understood and likely reflects a combination of the effects of several factors including haplotypic variation in the β-globin locus region, the action of genetic modifiers elsewhere in the genome, and a wide range of environmental factors (Weatherall, 2001; Sankaran et al., 2010).

Mapping genetic variants associated with SCD clinical phenotypes have largely been limited to candidate gene approaches and genome-wide association studies (Adams et al., 2003; Menzel et al., 2007; Lettre et al., 2008; Sebastiani et al., 2010; Solovieff et al., 2010; Thein, 2011; Steinberg and Sebastiani, 2012). One of the most characterized modulators of clinical expression of SCD is fetal hemoglobin (HbF). Higher HbF levels have been associated with reduced rates of acute pain episodes, leg ulcers, less frequent acute chest syndromes, and reduced disease severity (Akinsheye et al., 2011). Heritability for HbF is high (*h*^2^ approximately 0.60–0.89) and genetic factors that control its expression have been mapped (Sankaran et al., 2010). However, HbF levels have no clear association with other SCD clinical manifestations such as stroke and silent cerebral infarction, priapism, urine albumin excretion, and systemic blood pressure (Akinsheye et al., 2011). Several other genetic modifiers contributing to the variation in clinical expression of SCD have also been identified (Thein, 2011; Steinberg and Sebastiani, 2012), nonetheless most of the variation remains unexplained. Since disease in general involves differential expression (Emilsson et al., 2008; Cookson et al., 2009; Berry et al., 2010; Idaghdour et al., 2012), a systems genetics approach to map genetic variation associated with gene expression traits correlated with clinical phenotypes (Idaghdour and Awadalla, 2012) is likely to reveal regulatory variation modulating SCD.

We recruited HbSS and HbSC patients from a West African SCD cohort, using a two-phase sampling design, and generated a global map of gene expression variation and its genetic regulatory variation. Patients in the cohort were part of an established comprehensive clinical care program which includes an intensive socio-medical intervention program to impact the disease course (Rahimy et al., 2003). The vast majority of the children in the cohort show a severe clinical phenotype at some stage of the follow-up program. We set out to establish the extent of the effects of SCD relating to the Hb genotype and clinical follow-up on whole blood gene expression profiles, and also to identify genetic regulatory variation associated with gene expression traits. Furthermore, we test the hypothesis that gene expression variation associated with the disease or in response to clinical follow-up can be dependent on patient's regulatory genotypes, which in turn may explain inter-individual differences in disease severity. In so doing, we captured new genes associated with SCD clinical variation and identified genetic regulatory effects that explain a substantial percentage of transcriptional variation in SCD patients.

## Materials and methods

### Study population

Ethics approval for the study was granted by the Sainte-Justine Research Center Ethics Committee and by the Faculté des Sciences de la Santé of the University of Abomey-Calavi in Benin, West Africa. Informed consent was obtained for all participants in the study. Patients were part of a large cohort of SCD children longitudinally followed-up at the Centre de Prise en charge Médicale Intégrée du Nourrisson et de la Femme Enceinte atteints de Drépanocytose (CPMI-NFED), the National Institute of SCD Infants and Pregnant Women in Cotonou, The Republic of Benin. In total, 250 SCD patients aged between 6 months and 9 years old (mean age 4 years) and 61 healthy control siblings (not HbSS or HbSC and have at least one normal hemoglobin allele) were sampled under informed consent. A two-phase sampling design was used from February-December 2010 (Figure S1). The initial discovery phase included patients recruited mostly before the end of April, 2010, and the replication phase included patients that were recruited mostly between April 2010 and December 2010 (Figure S2). The distribution of SCD patients newly enrolled (E) and followed (FU), Hb genotypes, and sex were proportionate in both phases. The 61 healthy siblings were of roughly equal age (mean age 3 years) and sex proportions as the SCD patients, and were also recruited at the CPMI-NFED (Figure S2).

### SCD clinical status and severity score

The CPMI-NFED has an established comprehensive clinical care program that includes an intensive socio-medical intervention program to impact the disease course (Rahimy et al., 2003). The vast majority of children in the cohort show a severe clinical phenotype. SCD patients experiencing an acute event are labeled acute (A). For the purpose of the present study, two (2) sampling clinical categories were assigned to patients: patients sampled at enrolment into the program and in steady-state are labeled as entry (E), and patients already being followed at the SCD Center were labeled as FU. At the Center, most patients that are followed obtain a steady-state condition with general clinical improvement that involves increased velocity of linear physical growth and marked reduction in the frequency and severity of SCD-related acute events; however, some followed patients experience no such improvement.

Age-matched healthy siblings were recruited as controls (Ctls). Three quarters of our Ctls are heterozygous HbAS and 1/4 are homozygous HbAA. Only 14 probes were differentially expressed between HbAA and HbAS individuals at FDR 1%. Furthermore, none of the variance in the Ctls was explained by this effect as evidenced by variance component analysis and by the lack of clustering based on Hb genotype in PCA analysis (Figure S2). For these reasons, we grouped HbAA and HbAS individuals and used them as a control sample.

A quantitative SCD severity score (SV) was calculated using an online SCD severity calculator (http://www.bu.edu/sicklecell/projects/) (Sebastiani et al., 2007) where each patient was assigned a score based on their sex, Hb genotype, mean corpuscular volume (MCV), and white blood cell (WBC) counts. Ctls were assigned a score of 0.

### Sample preparation

The same collection procedure was followed for all samples in order to reduce technical heterogeneity. A total of 10 ml of peripheral whole blood was collected for each patient between 9:00 am and 2:00 pm and stored at −30°C. Shipment to Montreal was done at −20°C. Approximately 3 ml of this blood was collected for RNA work in TEMPUS blood RNA Tubes (Life Technologies); and approximately 5 ml of this blood was collected in EDTA tubes for DNA work; the remainder of the blood was used for complete blood counts using an automated KX-21 blood analyzer (Sysmex Corporation, Japan), identification of the hemoglobin phenotype by high-performance liquid chromatography (HPLC) and Capillary Electrophoresis, and thick smear analysis for parasetemia quantification. Total RNA was isolated using the TEMPUS RNA extraction kit (Life Technolgies) following the manufacturer's recommendations. A globin mRNA reduction step was performed using GLOBINclear-Human kit (Life Technologies). Total RNA extractions were quantified and quality was checked using the RNA 6000 Nano LabChip kit and 2100 Bioanalyzer (Agilent Technologies). Only samples of high RNA quality (Agilent's RNA Integrity Number >7.5) were retained for expression profiling. DNA samples were extracted using QIAamp DNA Kit (Qiagen). Quantity and quality was checked using Agilent's DNA 6000 Nano LabChip kit and the 2100 Bioanalyzer (Agilent Technologies).

### Genotyping the β-globin locus

Identification of the rs334 genotype and characterization of haplotype structure in the Hb locus was performed using Sequenom MassARRAY technology for 237 patients using 600 ng of genomic DNA and following the manufacturer's recommended protocols. Individuals with less than 75% call rate were excluded (Figure S2).

### Data sets

Four datasets were used for gene expression analyses: the discovery phase (126 SCD patients and 31 Ctls), the replication phase (124 SCD patients and 30 Ctls), combined dataset I (250 SCD patients and 61 Ctls), and combined data set II (160 SCD patients and 56 Ctls). Combined data set II includes only HbSS SCD patients excluding those sampled during acute crises. For the joint genotypic and gene expression data analyses, the combined dataset II (*n* = 173) included 120 SCD patients and 53 Ctls (Figure S1, Supplementary File 3).

### Gene expression profiling

Illumina's HumanHT-12 v4 BeadArrays were used to generate expression profiles of more than 48,000 probes using 500 ng of labeled cRNA for each sample following the manufacturer's recommended protocols. All expression data are available at NCBI Gene Expression Omnibus (GEO) under the series number GSE35007. The individual expression arrays are listed as GSM860207 through GSM860517. To minimize chip and batch effects, a randomized design was used. Hybridization was performed on two different dates and 4 samples from the first hybridization batch were re-hybridized with the second batch. These technical replicates clustered adjacent to one another in hierarchical analysis, indicating a negligible batch effect on the data. This was confirmed by testing for batch effect in the probe-by-probe analysis of variance. The expression intensities were averaged for each probe in the statistical analysis. The raw intensities were extracted using the Gene Expression Module in Illumina's BeadStudio software. Expression intensities were log2 transformed and quantile normalized using JMP Genomics v5.0 (SAS) after an outlier filtering procedure was applied. In total, 28,595 probes with expression at or above background levels averaged across all the arrays were retained for further analyses. These represent probes remaining after removal of 18,404 probe measurements that were considered to lay below background detection levels indicated by the inflection point in a plot of rank-ordered normalized intensities. Also, 427 probes overlaying SNPs included in the Illumina's OmniExpress BeadChip were removed from the analysis. Pathway and gene ontology analysis was performed using Gene Set Enrichment Analysis (GSEA) (Subramanian et al., 2005).

### Genome wide genotyping

Genome-wide genotyping data was generated for over 733,200 SNPs using Illumina's HumanOmni Express BeadChip arrays following manufacturer's protocols and extracted using the Genotyping Module in Illumina's BeadStudio software. Marker properties were calculated using PLINK (Purcell et al., 2007). Only SNPs with minor allelic frequency >5%, a call rate >99% and SNPs that are in Hardy-Weinberg Equilibrium (HWE) were included (*p*-value > 0.001). This resulted in a final set of 568,921 SNPs for further analysis. Global genotypic variation and ancestry was inferred using Eigenstrat (Price et al., 2006) and STRUCTURE (Pritchard et al., 2000); we detected limited population structure in our sample (Figures S2, S3). Insignificant population structure and limited genetic differentiation were also observed when 541 genotypes from a subset of genes known to influence hemoglobin levels (alpha-globin, G6PD, BCL11A, MYB, and HBS1L) were used in PCA and gene-wise Fst analysis to estimate the magnitude of genetic differentiation among the clinical statuses investigated and between them and the Ctls.

### Gene expression data analysis

All statistical analyses of the gene expression data were performed using JMP Genomics v5.0 (SAS), and SAS 9.3 (SAS). Principal Component analysis (PCA) and Variance Component analysis (VCA) of the gene expression data were performed such that the first three expression PCs (ePCs) were modeled either simultaneously or individually as a function of various effects in the data: Hemoglobin genotype, clinical status (E vs. FU vs. Ctls), sex, and pair-wise combination of fixed effects. SAS GLM was used to evaluate the magnitude and significance of differentially expressed probes. Probe-level differential expression analysis was performed using analysis of covariance. Variance was partitioned among the Hemoglobin genotype (Hb), clinical status effect, sex, and total blood cell counts (RBCs and WBCs) as covariates. The effects of date of sampling, phase (discovery vs. replication), age (in years), and gPCs were tested and found to be marginal. Pairwise contrasts (Hb genotype × Sex, Hb genotype × ClinStatus, and ClinStatus × Sex) also were evaluated and found to be insignificant. Results from the following full ANCOVA model are detailed in Figure 2: Expression = μ + Hb genotype + ClinStatus + Sex + WBC + RBC + ε.

The error ε was assumed to be normally distributed with mean equal to zero. The 3-way clinical status effect (E vs. FU vs. Ctls) was evaluated. A statistical significance threshold of 1% FDR was applied separately to each term in the analysis of covariance.

### eSNP mapping

Multiple linear regression analyses were performed using PLINK to test for significant associations between gene expression levels for each probe and SNP genotype. Only well-annotated, autosomal probes with validated chromosomal location and gene function based on the most recent annotation in NCBI and UCSC as of October 2011 were included for the association tests. In the process we aligned all probes to the reference genome (hg19), excluded ambiguous and all non-RefSeq probes, and removed 427 probes overlaying known SNPs from the analysis. This resulted in a total of 19,431 expressed probes that were tested for association with 560,675 SNPs. SNPs with a minor allelic frequency <5%, an exact HWE *P*-value < 0.001, or >1% missing data were excluded. We distinguished between local and distal associations based on the chromosomal location of the probe-SNP pair; a local association implicates a probe and a SNP located on the same chromosome while a distal association implicates a probe and a SNP located on different chromosomes. We applied Bonferroni correction for all eSNP associations in this study by accounting for both the number of SNPs and loci tested. Since 560,675 SNPs were tested for association with 19,431 probes, a genome-wide Bonferroni threshold for distal-associations corresponds to 0.05/(19,431 probes × 560,675 SNP) = 4.59 × 10^−12^ and for local associations to a Bonferroni threshold of 0.05/(19,431 probes × 200 SNPs) = 1.28 × 10^−08^ considering an average number of 200 SNPs tested against each probe.

In the multiple linear regression eSNP analysis, we tested for association between probe expression levels and SNP genotype while accounting for clinical status effect (ClinStatus), sex and blood cell counts (white and RBC counts, WBC and RBC), assuming that the error ε is normally distributed with a mean of zero, where:

$$\begin{array}{l} \text{Model 1}:\text{Expression}=\mu +\text{SNP}+\text{ClinStatus}+\text{WBC}\\ \;+\text{RBC}+\text{Sex}+\varepsilon \end{array}$$

The significant associations were compared to the associations reported in 12 published eQTL studies of peripheral blood or its derivatives at nominal *P*-values < 10^−7^. These published associations were accessed using the eQTL Browser (http://eqtl.uchicago.edu/cgi-bin/gbrowser/eqtl/) and compared to our results.

### Interaction effects

We tested for SNP-by-clinical status interaction effects using 7002 probes differentially expressed for the 3-way clinical status effect (E vs. FU vs. Ctls, FDR 1%) using combined data set II. For this analysis and to reduce the effect of outlier expression values, we further filtered the set of genotypes and included only SNPs with a minor allelic frequency <5%, an exact HWE test *P*-value < 0.001 and >1% missing data calculated in each of the sub-groups of patients separately (455,750 SNPs). This resulted in a final set of 455,750 SNPs tested against 7002 probes while accounting for clinical status effect (ClinStatus), sex, cell counts (white and RBCs, WBC and RBC) and including a term for SNP × ClinStatus in the model:

$$\begin{array}{l} \text{Model 2}:\text{Expression}=\mu +\text{SNP}+\text{ClinStatus}+\text{WBC}+\text{RBC}\\ \;+\text{Sex}+\text{SNP}\times \text{ClinStatus}+\varepsilon \end{array}$$

where ε is assumed to be normally distributed with a mean of zero.

A genome-wide Bonferroni correction was applied by accounting for both the number of SNPs and loci considered in this analysis. Since 455,750 SNPs were tested for association with 7002 probes, a genome-wide Bonferroni threshold for distal-associations corresponds to 0.05/(7002 probes × 455,750 SNP) = 1.57 × 10^−11^ and for local associations to a Bonferroni threshold of 0.05/(7002 probes × 200 SNPs) = 3.57 × 10^−08^ considering an average number of 200 SNPs tested against each probe.

To account for relatedness in our samples, we generated a matrix of pairwise relatedness estimates (IBD) for all possible pairs of individuals in our cohort. Only autosomal SNPs with a MAF >0.1, missingness of 0%, and that were not in linkage disequilibrium (*r*^2^ < 0.3) were included in estimating relatedness (final number of SNPs = 1992 SNPs). We used this matrix to estimate the random effects of relatedness in a Q-K mixed model framework using the GLIMMIX procedure in SAS (Yu et al., 2006). This procedure is computationally intensive and was applied only to the associations deemed initially statistically significant for the interaction effect prior to accounting for relatedness.

## Results

### Study design and case description

A total of 311 children from Cotonou, Benin, West Africa, were recruited for this study (Table 1, Figure S1). Here, we distinguish between two groups of SCD patients: those who were newly admitted into the program and were labeled as entry (E) and those sampled after being followed and were labeled as FU (see Materials and Methods for details). The initial discovery phase included 126 SCD patients recruited in early 2010, and the replication phase included 124 SCD patients recruited in late 2010. The distribution of clinical categories, Hb genotypes, and sex were proportionate in both phases (Figure S2). In addition, 61 healthy siblings with at least one normal hemoglobin allele and of similar age and proportions of sex were recruited at the CPMI-NFED (Table 1, Figure S2). SCD patients were also assigned a SV (Sebastiani et al., 2007) based on sex, Hb genotype, and cell counts. For all 311 participants, whole blood samples were collected, and complete blood cell counts (CBCs), genome-wide gene expression profiling and genome-wide genotyping were generated. Variabilities in RBC and WBC counts were treated as covariates in the analyses of variance.

**Table 1:** **Characteristics of study participants**.

**Dataset**	**Analysis**	***n***	**Sex**	**Hb genotype**			**ClinStatus**		
			**%F**	**HbSS**	**HbSC**	**Ctls**	***E***	***FU***	***A***
**Discovery**									
	Gene expression	157	0.49	99	27	31	70	38	18
	Genotyping	142	0.49	89	23	30	62	33	17
**Replication**									
	Gene expression	154	0.48	91	33	30	64	42	18
	Genotyping	129	0.49	68	32	29	53	31	16
**Combined I**									
	Gene expression	311	0.49	190	60	61	134	80	36
	Genotyping	263	0.48	151	54	58	110	64	31
**Combined II**									
	Gene expression	216	0.52	160	n/a	56	102	58	n/a
	Genotyping	173	0.55	120	n/a	53	79	41	n/a

Patient characteristics in the discovery phase, replication phase, and combined datasets I and II. Combined data set II includes only HbSS SCD patients excluding those sampled during acute crises. Approximately, equal numbers of girls and boys were sampled and approximately 2/3 of the SCD patients have the HbSS genotype. Controls have either the HbAS or HbAA genotype. Mean age of the patients and controls was 4 and 3 years, respectively. Sampling was done between February 2010 and December 2010. The numbers of individuals gene expression profiled (Illumina HumanHT-12) or genotyped (Illumina OmniExpress) in each phase are shown. Each main variable Clinical status: (Entry, E; Follow-up, FU; Acutes, A; and Controls, Ctls; Sex (% F, % Female); and Hb Genotype: (HbSS; HbSC; Ctls, C) was sampled in equal proportions in the discovery phase, replication phase, and in the combined datasets. n/a, non-applicable.

### Differential gene expression analysis—discovery phase

**Figure 1:**
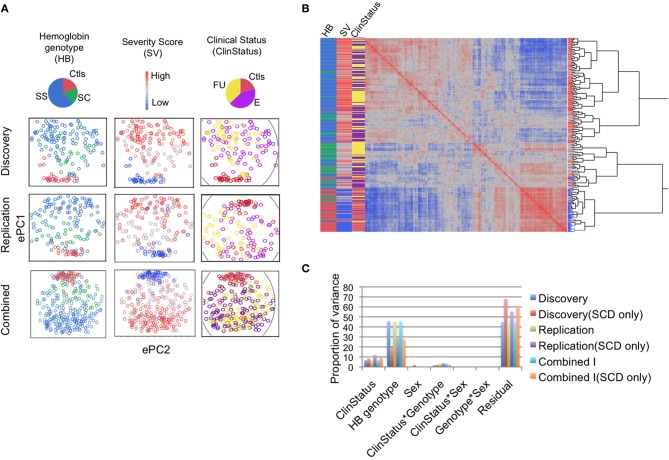
**Sickle cell disease impacts gene expression genome-wide. (A)** The first two expression principal components (ePC) from PC analysis of the discovery and replication phase samples, and in the combined dataset. Individuals are coloured according to Hb genotype (HbSS, blue; HbSC, green; and Controls, red), SCD severity score (SV, red to blue indicates high to low severity) and clinical status effect (ClinStatus, yellow; E, purple, Ctls, red). **(B)** One-way hierarchical clustering of the genome-wide gene expression correlation matrix for the combined dataset (*n* = 311). The heat map shows the clustering of individual expression profiles based on similarity. The highest level of clustering is observed for the Hb genotype effect followed by SCD severity score. **(C)** Variance component analysis (VCA) of the first three expression PCs (ePC1-3) explaining 36, 37, and 37% of the total variance in the discovery, replication, and in the combined dataset. The two main variables that explain this variance are Hb genotype and clinical status effect. The proportion of the variance explained by each variable is similar in the discovery, replication and combined datasets. VCA of SCD patients alone shows that the proportion of the variance explained by clinical status was similar to that when the controls were included but the proportion of the variance explained by Hb genotype dropped by 25–50%. See also Figure S4.

Analysis of gene expression shows that SCD has substantial influence on the whole blood transcriptome. In the discovery phase (*n* = 157), unsupervised hierarchical clustering analysis of the genome-wide gene expression correlation matrix revealed that individual gene expression profiles cluster largely according to Hb genotype, SCD SV, and clinical status (E vs. FU vs. Ctls; Figures 1A,B). PCA revealed the presence of strong correlation structure in the data such that the first three expression principal components (ePC1-3) explain over a third of the total variance (Figure S4). VCA of the first three ePCs further confirms the substantial effect of Hb genotype (explaining 45.6% of the variance) followed by clinical status (explaining 7% of the variance) (Figure 1C). Variance of ePC1 was explained primarily by Hb genotype (>70%) while ePC2 and 3 were dominated by the effect of clinical status, explaining 20% of the variance of each PC; sex and interaction effects had negligible effects on the variance (Figure S4). Repeating this analysis with only SCD patients (*n* = 126) revealed that a third of the variance (31%) was captured by the first three ePCs, with Hb genotype and the FU effect explaining 19.5 and 8.6% of the variance, respectively (Figure 1C).

**Figure 2:**
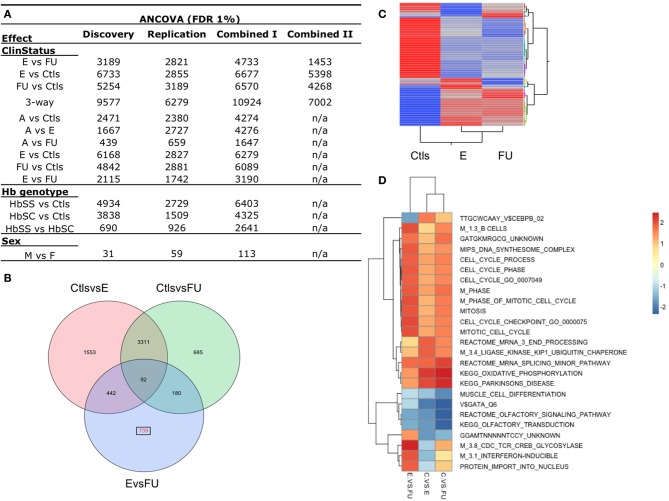
**Differential gene expression between SCD disease status. (A)** Number of differentially expressed probes for the following effects: SCD clinical status (E, Entry; FU, Follow-up; Ctls, Controls; A, Acute), Hb genotypes (HbSS, HbSC, Ctls), and between sexes (M, males; F, females). The 3way-ClinStatus effect is between E vs. FU vs. Ctls. These results were obtained from an analysis of covariance (ANCOVA, FDR 1%) of the discovery, replication and combined datasets I and II and accounts for sex and total blood cell counts (RBC and WBC). **(B)** Venn diagram of the 7002 differentially expressed probes for the 3-way clinical status effect in the combined data set II. In red, 735 probes are shown to be differentially expressed uniquely between E vs. FU SCD patients. **(C)** Two-way hierarchical clustering of the mean expression levels for the 7002 differentially expressed probes in the combined data set II for each group of patients (E, FU, Ctls) is shown. Mean expression from this class of genes cluster controls from SCD entry and follow-up patients. **(D)** Gene Set Enrichment Analysis (GSEA) was performed for each contrast of the clinical status effect using the combined dataset II. This analysis identified biological pathways and sets of individual genes that are significantly enriched in each contrast. Selection of the most distinctive significantly enriched pathways between entry and follow-up groups is shown. Cells are colored by their respective Normalized Enrichment Scores for a given contrast. See also Figure S6.

Next, we evaluated the magnitude and significance of differentially expressed genes between SCD clinical status and Ctls. Given that a fraction of the variation in ePCs is likely due to differences in the proportion of cell types between SCD patients, we performed a probe-by-probe analysis of covariance (ANCOVA) of the discovery sample that accounts for total blood cell counts (RBC and WBC counts), in addition to sex, and genetic ethnicity using individuals' scores at significant genotypic PC axes (see Materials and Methods for details). This analysis revealed significant differences between SCD patients (E and FU) and Ctls with a quarter of the transcriptome being differentially expressed for the 3-way clinical status effect at 1% False Discovery Rate (FDR) (Figure S5). Thousands of genes were also significantly differentially expressed between Hb genotypes (HbSS, HbSC, Ctls) while minor differences were observed between sexes (Figure 2A) and no effect of the genome-wide genotypic ethnicity effect (gPCs) was detected (Figure S5). Since meaningful population structure in the sample was not observed (Figures S2, S3) and since no probes were significant for the gPC effect (FDR 1%) (Figure S5), genetic ancestry is unlikely to contribute significantly to the observed gene expression differences in our sample.

### Replication of differential expression among SCD patient groups and controls

To test the consistency of the patterns of gene expression differentiation observed in the discovery phase, we performed the analyses described above on the replication group (*n* = 154) and the combined dataset (combined dataset I, *n* = 311, see Table 1) and observed similar results (Figures 1, 2A, S4, S5, Supplementary File 5). Unsupervised analysis identified similar clustering by Hb genotype, SCD SV and the clinical status effect (Figures 1A,B), with Hb genotype and the clinical status effect explaining 45.2 and 6.9% of the variance of the first three ePCs in the replication phase, respectively (Figure 1C, Figure S4). When only SCD patients were included, Hb genotype and the FU effect explained 28.8 and 12.1% of the variance in the first three ePCs, respectively (Figure 1C). The magnitude and significance of differentially expressed probes for the clinical status and Hb genotype effects were highly consistent in both replication and discovery phases (Figures 2A, S5).

Next we focused on 160 SCD HbSS patients and 56 Ctls (combined data set II, *n* = 216, see Table 1) to characterize the transcriptional signatures associated with SCD clinical status and follow-up. HbSC individuals were excluded from this analysis given their small sample size relative to the HbSS group. SCD patients undergoing an acute event were also excluded to focus on the steady state of the disease. An ANCOVA of this dataset accounting for sex and total cell counts revealed that over seven thousand probes were significantly differentially expressed (1% FDR) for the clinical status effect (Figure 2A) and 739 probes for the FU effect (Figure 2B). The effect of clinical status is visually shown in a heat map generated using a 2-way hierarchical clustering of per-group mean expression levels of differentially expressed probes (Figure 2C). The supervised and unsupervised gene expression analysis of both the discovery and replication samples documented the relative contribution of Hb genotype and clinical status to the transcriptional variation observed in a West African SCD cohort and characterized the effects taking place after clinical follow-up. These analyses show that SCD has a substantial influence on whole blood transcriptome with Hb genotype and clinical status explaining the majority of the variation.

### Identification of biologically relevant pathways through enrichment analysis

In order to identify the biological pathways subject to the effects of differential expression associated with SCD disease and clinical follow-up, GSEA (Subramanian et al., 2005) was performed using the results of differential expression analysis described above for the discovery, replication and combined datasets (I, II). We focused on gene sets with Normalized Enrichment Scores (NES) greater than 0.25 in either E or FU relative to the Ctls as shown in Figure 2D. This analysis showed that the strong modulation of the transcriptome implicates pathways affecting core circulating cell functions. A strong activation of pathways associated with B-lymphocytes development, stress (glucocorticoid, interferon and oxidative phosphorylation associated pathways) and cell proliferation in E compared to the FU group was observed. We also note a significant up-regulation of genes specific to platelet function and erythrocyte membrane in SCD individuals relative to the control and to a lesser extent in FU relative to the E group.

### The genetic architecture of transcript abundance in SCD

**Figure 3:**
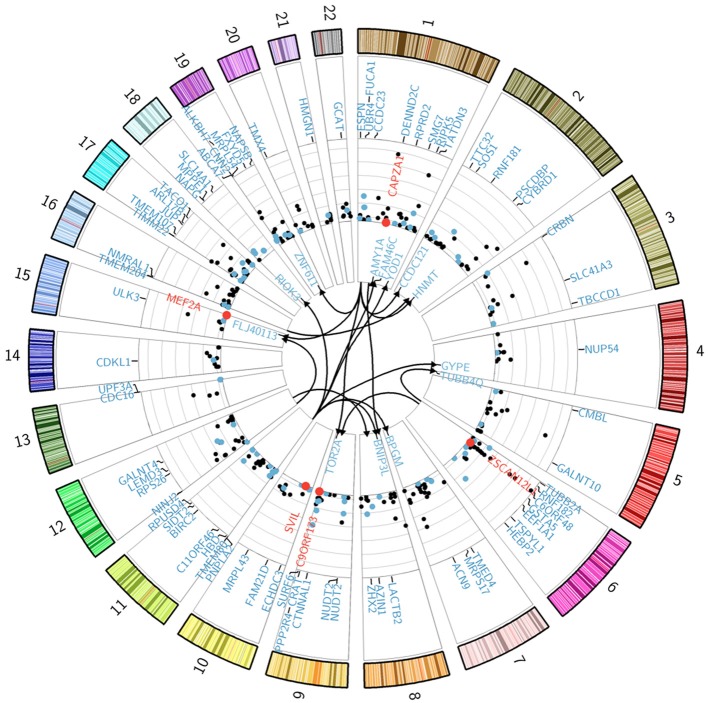
**Genetic regulation of gene expression in SCD patients**. The Circularized Manhattan plot shows genome-wide significant SNP-probe associations for the analysis that used the combined II dataset. Bonferroni correction for multiple testing was applied to all of our analyses with a genome-wide significance threshold of *p* < 0.05/(19,431 probes × 200 SNPs) = 1.28 × 10^−08^ (NLP = 7.89) for local associations in model 1 and *p* < 0.05/(19,431 probes × 560,675 SNP) = 4.59 × 10^−12^ (NLP = 11.34) for distal-associations in model 1; while model 2 thresholds were *p* < 0.05/(7002 probes × 200 SNPs) = 3.57 × 10^−08^ (NLP = 7.45) for local associations and *p* < 0.05/(7002 probes × 455,750 SNP) = 1.57 × 10^−11^ (NLP = 10.80) for *distal*-associations. Distal associations are shown in the center of the plot. All genes involved in an interaction effect are differentially expressed and shown in red. eSNP genes from model 1 that are differentially expressed for the clinical status effect are shown in blue. The y-axis of the Manhattan plot indicates significance values (−log^10^ *p*-values) for the local-associations. Genes under eSNP control that are not differentially expressed for the clinical status effect (in the ANCOVA analysis at FDR 1%) are shown in black. See also Table S1.

The genetic architecture of transcript abundance in SCD was investigated through genome-wide association analysis of gene expression traits in SCD patients and Ctls. Given the high degrees of correlation in the results of gene expression analyses for the discovery and replication phases and to increase mapping power, we performed the analysis on a subset of the combined dataset II (*n* = 173: 120 HbSS SCD patients and 53 Ctls) for which both gene expression and genotypic data were available. The expression data for the combined dataset was re-normalized in order to minimize potential batch effects, resulting in a final set of 19,431 probes tested against 560,675 SNP genotypes using multiple regression analyses and applying Bonferroni correction for multiple testing. For local associations, the genome-wide significance threshold corresponds to testing on average 200 SNPs against each probe. For distal associations, each probe was tested against each of the 560,675 SNPs. We ran a model that accounted for participant clinical status (E, FU, and Ctls), total blood cell counts (RBS, WBC), and sex (Model 1, see Materials and Methods). Three hundred and ninety genome-wide significant peak SNP-probe associations were identified corresponding to 371 local and 19 distal effects (Figure 3). These associations explain on average a third of the variance in transcript abundance.

### eSNP-by-clinical status interactions

**Figure 4:**
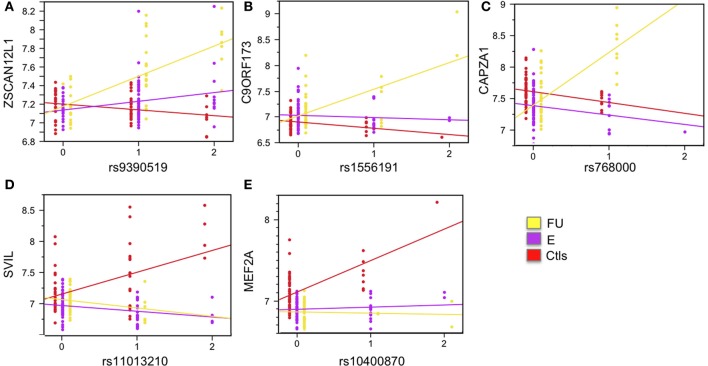
**Examples of significant SNP-by-clinical status interaction effects**. Five SNP-by-clinical status interaction effects are shown. All are local eSNP interactions. Expression levels are shown on the y-axis, and SNP genotype on the x-axis. The eSNP interaction involving gene zinc finger and SCAN domain containing 12 pseudogene 1 (ZSCAN12L1) is shown in **(A)**; chromosome 9 open reading frame 173 (C9ORF173) is shown in **(B)**; capping protein (actin filament) muscle Z-line, alpha 1 (CAPZA1) is shown in **(C)**; supervillin (SVIL) is shown in **(D)**; and myocyte enhancer factor 2A (MEF2A) is shown in **(E)**. Linear regression for each group is plotted and colored: yellow for follow-up, FU; purple for entry, E; and red for controls, Ctls. See also Figure S7.

Differential expression analysis revealed 7002 probes significantly differentially expressed (1% FDR) for the clinical status effect. In order to identify which of these genes are under strong genetic regulatory effects that are dependent on clinical status we tested for the SNP-by-ClinStatus interaction effect by including it as term in Model 1 (See Materials and Methods for details). Bonferroni correction for multiple testing in this analysis was applied. The markers included in this analyses were limited to SNPs with MAF >5% in each clinical group (See Materials and Methods for details). This analysis revealed 11 significant interaction effects, six of which remained genome-wide significant after accounting for relatedness in the entire sample using a Q-K mixed model (Yu et al., 2006) (see Materials and Methods for details): ZSCAN12L1 (*p*-value = 4.26 × 10^−10^), C9ORF173 (*p*-value = 8.94 × 10^−9^), CAPZA1 (*p*-value = 1.33 × 10^−8^), SVIL (*p*-value = 2.41 × 10^−8^), MEF2A (*p*-value = 1.69 × 10^−8^), and C1ORF88 (*p*-value = 5.42 × 10^−9^). These interactions are visualized in Figures 4, S7. Figures 4A–C shows three local eSNP interaction effects where higher expression levels of the corresponding gene in the FU group relative to both the E group and the Ctls is driven by the minor allele of the eSNP in question. Figures 4D,E shows two associations where the higher expression levels in the Ctls relative to SCD patients is observed only in the presence of the minor allele for the corresponding eSNP.

## Discussion

Here, we characterized the transcriptomes of SCD patients. We first identified the extent of gene expression variation in SCD patients that is explained by clinical phenotypes and measured the magnitude and significance of gene expression differences for SCD clinical status in an initial discovery phase. The unsupervised analysis of gene expression profiles shows that SCD has substantial influence on the human transcriptome, explaining over a third of the total variance, followed by Hb genotype and SCD clinical status. Significant differences in gene expression profiles between SCD clinical status and Ctls were also observed, with over a quarter of the transcriptome being differentially expressed. We replicated these findings in a replication cohort.

Using GSEA, we identified and replicated biological pathways involved in the clinical course of SCD. This analysis shows that the strong modulation of the transcriptome implicates pathways affecting core circulating cell functions. Enrichment analysis also showed that several biological pathways previously reported to be associated with SCD (Jison et al., 2004) are subject to differential expression between the three clinical groups. Furthermore, we observed strong activation of pathways associated with B-lymphocyte development, stress and cell proliferation in the E compared to the FU group. Enrichment of genes that were uniquely differentiated between the E and FU patients identified a significant up-regulation in B-lymphocytes expressing phosphorylated CD5, B-cell Receptor Signaling and upstream regulation of B-cells by PAX5. PAX5 expression has been shown to increase the quantity and the commitment of B cells (Horcher et al., 2001). These observations reflect perturbed cellular profiles in the E groups and more stable profiles after clinical FU. Furthermore, markers of mitosis, cell cycle and DNA synthesis were identified in the analysis on combined data set II and likely suggest a more stable state of blood cells in the FU group in general. The strong interferon related signature also suggests a more perturbed and potentially more pathogenic state of blood cells prior to clinical follow-up. The overexpression of activated B lymphocyte markers in the E group tends to point in that same direction. Previous studies have shown that changes in B cell function occurs during vaso-occlusive crisis (VOC) in patients with SCD (Venkataraman and Westerman, 1985). Thus, follow-up of SCD patients may act on these pathways. We also note a significant up-regulation of genes specific to platelet function and erythrocyte membrane in SCD individuals relative to the control group and to a lesser extent in FU relative to the E group. Activation of platelets in SCD patients was previously associated with clinical complications such as vasculopathy (Raghavachari et al., 2007) and hemolysis-associated pulmonary hypertension (Villagra et al., 2007). We observed a strong inflammatory response signature in Acute patients consistent with the processes induced during SCD crises events such as VOC (Musa et al., 2010).

We characterized the genetic architecture of transcript abundance in SCD patients and Ctls and identified 390 genome-wide significant peak SNP-probe associations. Four genes that are associated with an eSNP were previously associated with SCD phenotypes in reported association studies (Table S1). Almost half of the eSNP genes (150 eSNP genes) that we identified overlapped with previously reported significant eQTL associations (Table S1). Out of these, 58 were exact SNP-gene eSNP pairs. The overlap between our distal eSNP associations and those published in a recent paper that examined the effects of trans eQTLs as putative drivers of disease (Westra et al., 2013) identified three distal eSNPs (rs11171739, rs10493008, and rs6489721) in our SCD cohort that were also associated with genes in complex traits. Although the SNP-gene associations in our study were not exact matches with those reported in the (Westra et al., 2013) paper, it is possible that these 3 trans eSNPs are drivers for SCD related phenotypes.

Differential expression analysis revealed thousands of genes differentially expressed between clinical categories. We identified 11 eSNP interaction effects that are dependent on clinical status for this class of genes, six of which remained genome-wide significant after accounting for relatedness in the entire sample using a Q-K mixed model: *ZSCAN12L1*, *C9ORF173*, *CAPZA1*, *SVIL*, *MEF2A*, and *C1ORF88*. These genes represent novel SCD associations, form an interacting network generated using Ingenuity Pathway Analysis (www.ingenuity.com) (Figure S8) and have some overlapping clinical manifestations, particularly with respect to cardiovascular disease.

For example, *CAPZA1*, capping protein (actin filament) muscle Z-line alpha 1, is a gene located on chromosome 1 that encodes the alpha subunit of the barbed-end actin binding protein (Kuhlman and Fowler, 1997). *CAPZA1* has recently been associated with blood pressure variation in a meta-analysis of GWAS (Kato et al., 2011). In our study, we see an interaction between cis-acting eSNP rs768000 and clinical status such that SCD patients with the minor allele have higher expression of *CAPZA1* when they are followed-up. The *SVIL* gene encodes isoforms of supervillin, a protein that has been associated with KIRD2DL that regulates the inhibitory signal of natural killer cells that recognize MHC class I molecules (Liu et al., 2011). Recently, a study suggested an inhibitory role for SVIL in platelet adhesion and arterial thrombosis using human GWAS and mice knockout approaches (Edelstein et al., 2012). Edelstein et al. (2012) identified that platelets express *SVIL*; platelet thrombus formation is associated with human *SVIL* variants and low *SVIL* expression. We show a local association between *SVIL* gene expression and a SNP on chromosome 10, rs11013210. In our study, Ctls have a significant increase in *SVIL* gene expression when they have the minor allele for the SNP rs11013210. Finally, the *MEF2A* gene encodes a protein that is a DNA-binding transcription factor that activates many muscle-specific, growth factor-induced, and stress-induced genes (Zhao et al., 2012). Defects in this gene have been associated with autosomal dominant coronary artery disease 1 with myocardial infarction (Liu et al., 2012). *MEF2A* affects the proliferation, migration and phenotype of vascular smooth muscle cells (Zhao et al., 2012; Papait et al., 2013). In our samples, we see a local association between *MEF2A* gene expression and SNP rs10400870 genotype on chromosome 15 that is dependent on clinical status. Ctls have a significant increase in *MEF2A* gene expression when they have the minor allele for rs10400870.

In an attempt to estimate the contribution of the genetically controlled fraction of transcript abundance on the association between expression traits and clinical FU categories, we compared the effect of clinical status on global gene expression before and after conditioning for the eSNP effect on transcript abundance. To run this test we used the combined dataset II (focusing on the SNP effect and ClinStatus within the HbSS group) and applied it to all expressed genes. We used a full ANCOVA model with and without the SNP effect and applied the same stringent filtering criteria as described for the analyses above. We extracted *p*-values for the ClinStatus effect for all the genes and contrasted the two models. This comparison revealed overall a relatively high degree of correlation (Figure S9) suggesting that quantitatively most of the transcriptional signal differentiating the clinical categories is robust. However and as shown in Figure S9, we observe a global shift in the significance values toward lower values once genotypic effects are accounted for. To further show this trend, we limited our comparison to genes differentially expressed at Bonferroni level for the ClinStatus effect in at least one of the two tested models (*n* = 2188 genes). After fitting the genotype effect on each of these genes, 41% (*n* = 905) remain significant for the ClinStatus effect and 58% (*n* = 1275) are no longer significant. It is also worth noting that directionality of the effect between the two models was consistent for all tested genes. Moreover and as expected, our top interacting genes (based on *p*-values for the SNP × ClinStatus interaction effect) were among the list of genes that were subject to the highest drop in statistical significance for association with ClinStatus after fitting the eSNP effect. Taken together, these results suggest that transcript abundance of over half of expressed genes associated with clinical follow-up is under significant regulatory genetic effect.

Using eQTL approaches, transcriptional genotype-by-environment interactions have previously been reported in humans (Smirnov et al., 2009; Romanoski et al., 2010; Barreiro et al., 2012; Idaghdour et al., 2012) but mostly using *in vitro* systems (with the exception of Idaghdour et al., 2012). Here we demonstrate the existence of these effects *in vivo* in SCD. The genes implicated in these interactions show differential eSNP effects depending on SCD follow-up status. These interactions show how the genetic control of gene expression through allelic variation is likely to impact processes implicated in the response to SCD as well as to clinical follow-up programs.

In summary, using a two-stage sampling design, we identified and replicated a strong transcriptional signature of the effect of follow-up in SCD patients that implicates core biological pathways involved in the pathobiology of the disease. We have provided a genome-wide picture of regulatory variation *in vivo* in SCD patients and highlighted genotype-by-clinical status interaction effects that likely contribute to the clinical heterogeneity observed in SCD patients including those enrolled in SCD clinical follow-up programs. These results further our understanding of the transcriptional events occurring in SCD patients and their genetic regulatory control. The genetic and transcriptional markers reported here can potentially guide follow-up programs. These markers detected in whole blood, a readily and ethically accessible source of biological material in children, will be particularly useful in populations where the disease is most prevalent.

## Accession numbers

All expression data are available at NCBI GEO under the series number GSE35007. The individual expression arrays are listed as GSM860207 through GSM860517.

## Author contributions

Philip Awadalla and Mohamed C. Rahimy conceived the study. Mohamed C. Rahimy followed the SCD patients and oversaw characterization of SCD patient clinical categories. All hematological analysis was performed at the NSCDC under Mohamed C. Rahimy's direction. Philip Awadalla, Elias Gbeha, Selma Gomez, Jacklyn Quinlan, Mohamed C. Rahimy, Ambaliou Sanni, and Youssef Idaghdour collected the samples. Elias Gbeha, Jacklyn Quinlan and Youssef Idaghdour processed the samples and performed the genomic experiments. Vanessa Bruat, Thibault de Malliard and Jean-Christophe Grenier provided bioinformatics support for statistical analysis of the data by Philip Awadalla and Jacklyn Quinlan. Jean-Philippe Goulet performed enrichment analysis. Philip Awadalla, Youssef Idaghdour and Jacklyn Quinlan wrote the paper.

### Conflict of interest statement

The authors declare that the research was conducted in the absence of any commercial or financial relationships that could be construed as a potential conflict of interest.
