# Supplementary material for: Genomic architecture of sickle cell disease in West African children
Source: Front Genet. 2014 Feb 14;5:26. doi: 10.3389/fgene.2014.00026 (PMC3924578; doi:10.3389/fgene.2014.00026)
Supplement: Supplementary file 1 [file DataSheet1.DOC]

**Supporting Information**

**Genomic Architecture of Sickle Cell Disease**

- **in West African Children**
- **Jacklyn Quinlan^1,2,5^, Youssef Idaghdour^2,5^, Jean-Philip Goulet^2^, Elias Gbeha^2^, Thibault de Malliard^2^, Vanessa Bruat^2^, Jean-Chistophe Grenier^2^, Selma Gomez^3^, Ambaliou Sanni^3^, Mohamed Cherif Rahimy^4*^, Philip Awadalla^2*^**

^1^School of Public Health, Department of Social and Preventive Medicine, Faculty of Medicine, University of Montreal, Montreal, Quebec H2V 4P3, Canada

^2^Sainte-Justine Research Center, Department of Pediatrics, Faculty of Medicine, University of Montreal, Montreal, Quebec H3T 1C5, Canada

^3^ Laboratoire de Biochimie et Biologie Moléculaire, Faculté des Sciences et Techniques, Université d’Abomey-Calavi, Cotonou, RP, Benin

^4^ Centre de Prise en charge Médicale Intégrée du Nourrisson et de la Femme Enceinte atteints de Drépanocytose, Faculté des Sciences de la Santé, Université d’Abomey-Calavi, Cotonou, RP, Benin

^5^These authors contributed equally to this work.

^*^Correspondance: philip.awadalla@umontreal.ca, and mrahimy@bj.refer.org

**Content**

**Figures S1-S5**

**Tables S1**

**Legends for Supplementary Files 1-4**

**Supplementary Figures**

**
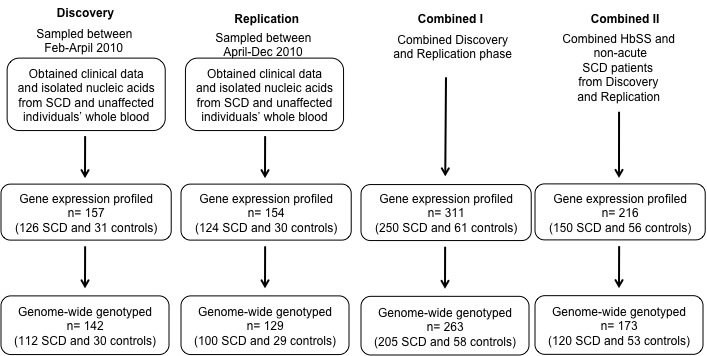
**

**Figure S1.** Diagram of sampling procedure for each phase. In each phase, the total number of SCD patients and controls that were genome-wide gene expression profiled and genome-wide genotyped for expression and eSNP analyses are detailed.

**
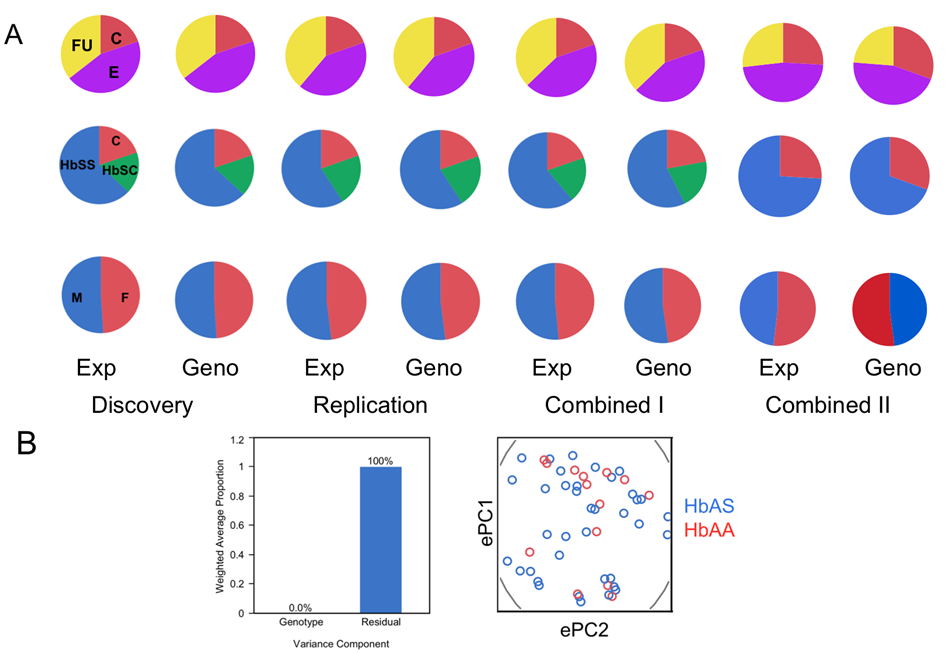
**

**
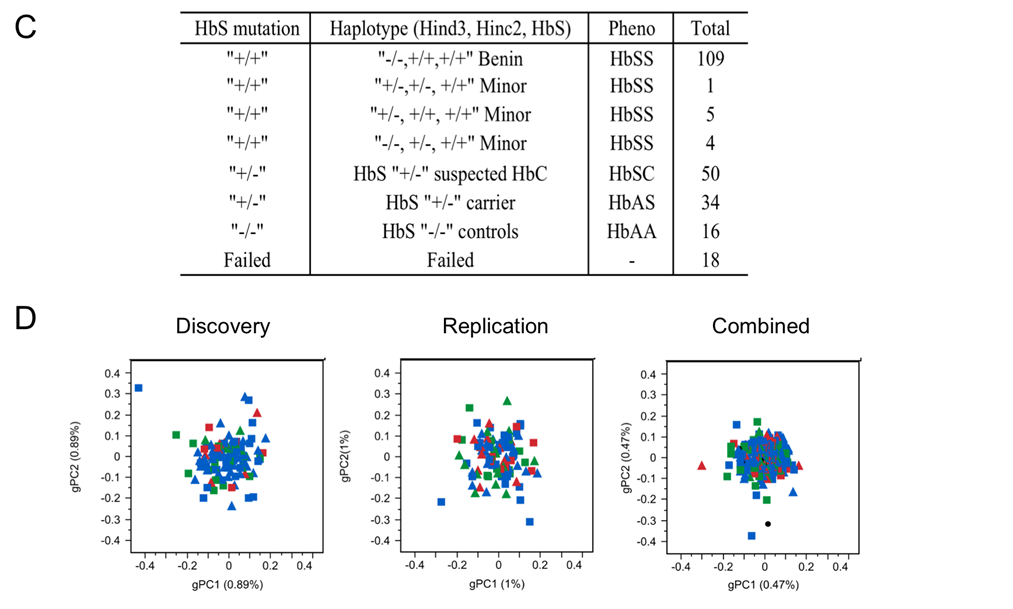
**

**Figure S2.**

**A) Patient Characteristics.**

Pie charts of patient characteristics in the discovery phase, replication phase, and combined I and II datasets for genome-wide gene expression analysis (Exp) and genome-wide genotyping analysis (Geno). Each main variable (Clinical status: Entry (E), Follow-up (FU), and Controls (C); Hb Genotype: HbSS, HbSC, Controls (Ctls, C); and Sex: Female (F), Male (M)) was sampled in equal proportions in the discovery phase, replication phase, and in the combined datasets.

- **B) Variance component analysis of expression principal componennts in the controls.** Variance component analysis for the Hb genotype effect (HbAS vs HbAA) in the controls on the first three expression principal components (ePC1-3) explains zero percent of the total variance in the combined dataset (A), and PC analysis identified a lack of clustering based on Hb genotype (B).

**C) Genotyping of the HbS and β-globin haplotypes**

Genotyping of the SCD HbS mutation (rs334) (A>T) and characterization of haplotypic structure in the Hb locus. Genotypic data was generated for 237 individuals using Sequenom MassARRAY technology. The haplotypes were constructed based on SNP genotypes at the RFLP sites for Hind3 and Hinc2 (+ and – signs indicate when the site is cut or nor, respectively)^1^. 109 patients had the HbSS genotype and were assigned the Benin haplotype. There were 10 patients with the HbSS genotype that had other haplotypes. Fifty patients had the HbSC genotype, 34 had the HbAS genotype, and 16 were HbAA controls. Genotyping failed for 18 individuals.

**D) Ancestry analysis for the discovery phase, the replication phase and the combined dataset.** Ancestry analyses of 119, 104 and 235 unrelated individuals from the discovery phase, replication phase and the combined dataset I using 485,000 genotypes. No obvious population structure was observed with all genotypic principal components (gPCs) explaining 1% or less of the total variance. The plots show the first two gPCs for phase1, phase2, and the combined dataset. No correlation was observed between gPCs and clinical status, sex (males,squares; females, triangles), or Hb genotype (blue, HbSS; green, HbSC; red, controls).

A

**
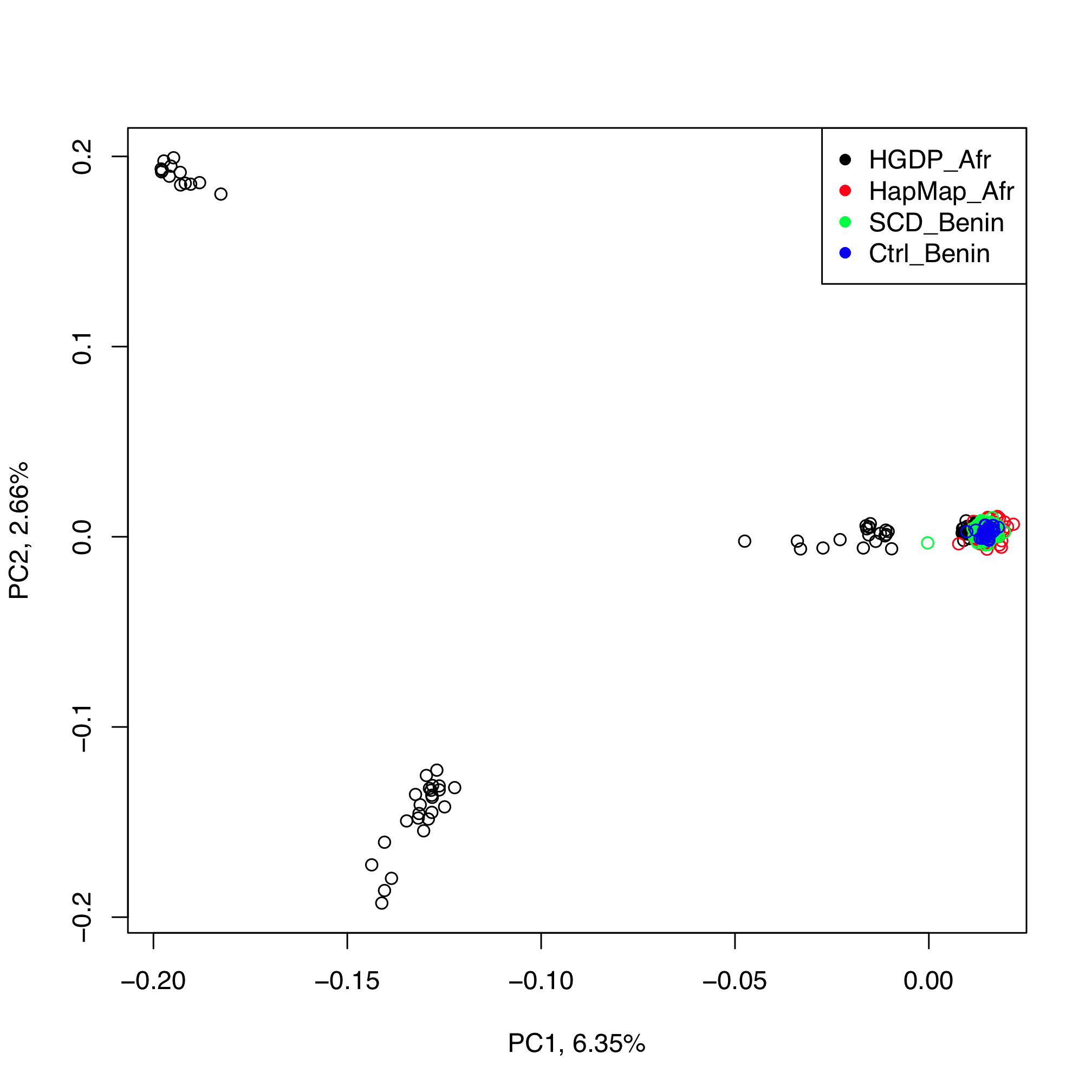
**

B

**
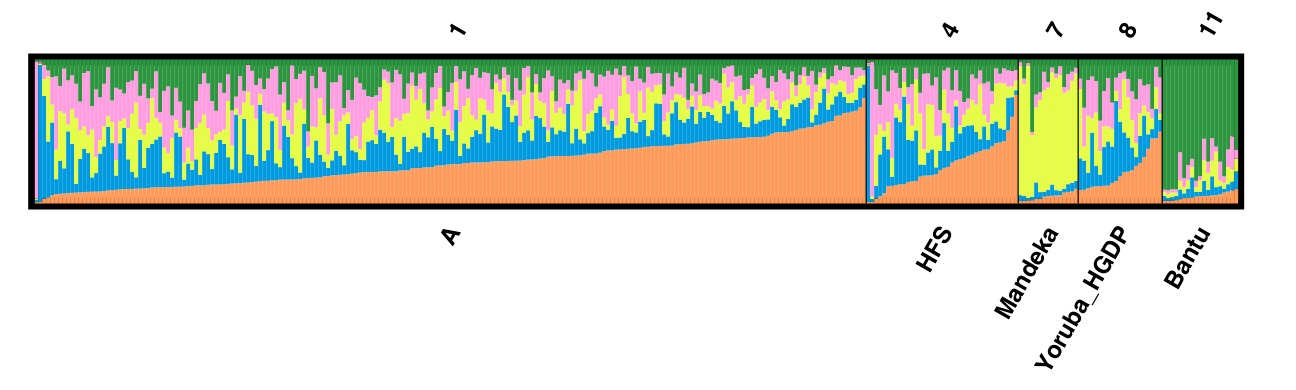
**

**Figure S3.**

**A) Ancestry analysis** of our cases (SCD_Benin) and controls (Ctrl_Benin) along with Yuruba (HapMap_Africa), Bantu, Biaka, San, Mandenka, and Mbuti samples from HGDP was performed using *Eigenstrat*. The genotypic PCs of the two major axes of variation are displayed.

**B) Structure analysis** for unrelated SCD patients (A) and controls (HFS), as well Mandeka, Yoruba, and Bantu from the Human Genome Diversity Project (HGDP). Population structure was infered using the program Structure. Any SNP with 1% missing data was removed. Our data set was merged with HGDP, and only overlapping SNPs were kept (n= 354434). A random set of 5000 SNPs were chosen for the analysis. Only unrelated SCD patients were included. K=5 and admix. A= SCD patients, HFS= controls

The results from (A) and (B) show homogeneity of the cohort relative to other ethnic groups in Africa and limited genetic ethnic differences between cases and controls.

**
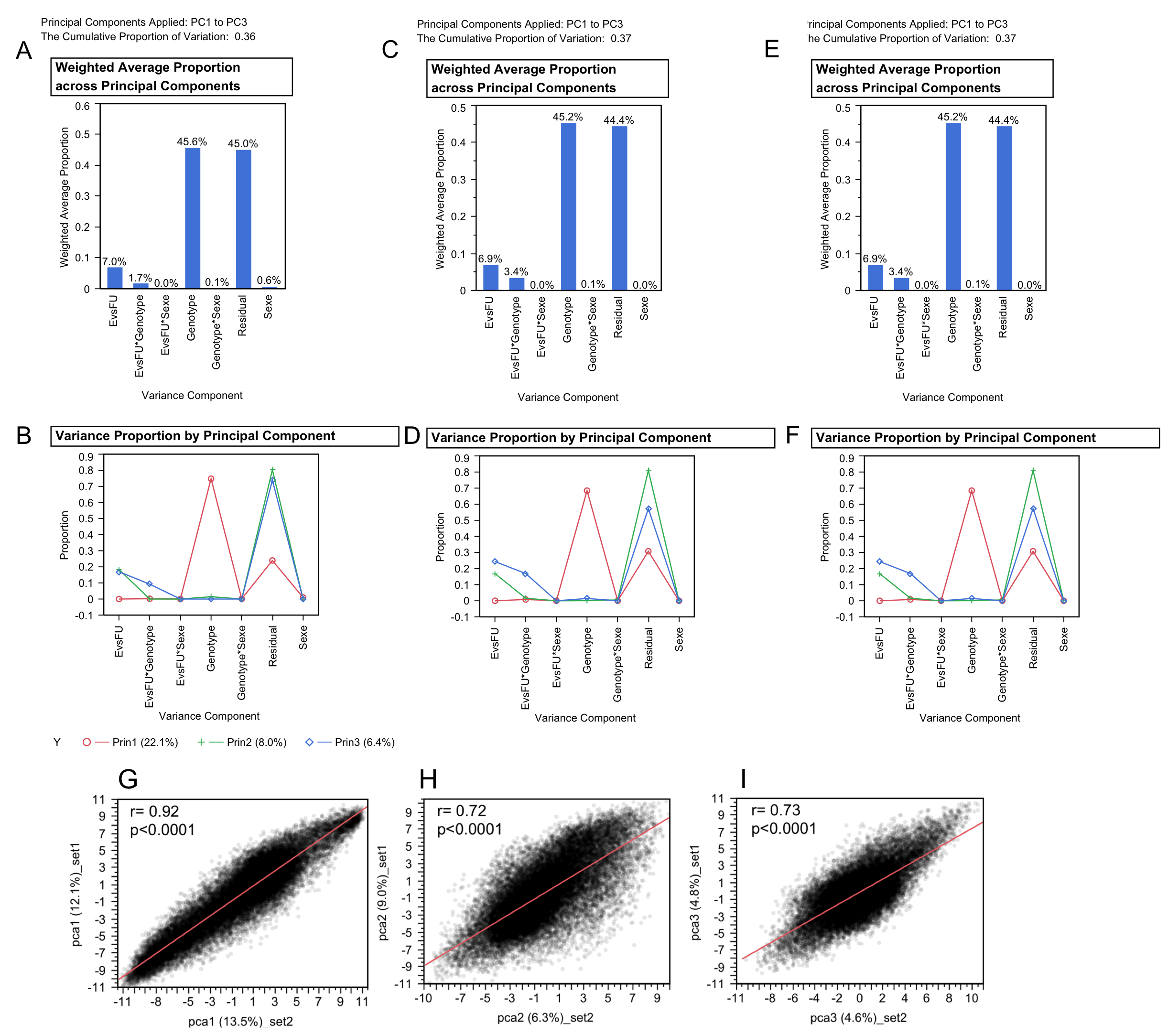
**

**Figure S4.**

**A-F Variance component analysis of expression principal components.** Variance component analysis on the first three expression principal components (ePC1-3) explains over a third of the total variance in the discovery phase (A and B), the replication phase (C and D), and in the combined data set (E and F). ePC1 is primarily explained by Hb genotype (phenotype), while ePC2 and ePC3 are driven by clinical status (EvsFUvsC) and follow-up (EvsFU) in all three datasets.

**G-H Pearson correlation of Principal Components (PC) scores for each gene in the discovery (set 1), and replication phases (set 2).** PCA of the gene expression data was performed for the discovery and replication. Principal component scores of all genes for each phase were contrasted. This analysis shows high correlation between discovery and replication phases. The correlations are shown for the first three ePCs; in G) ePC1, H) ePC2, and I) ePC3.

-
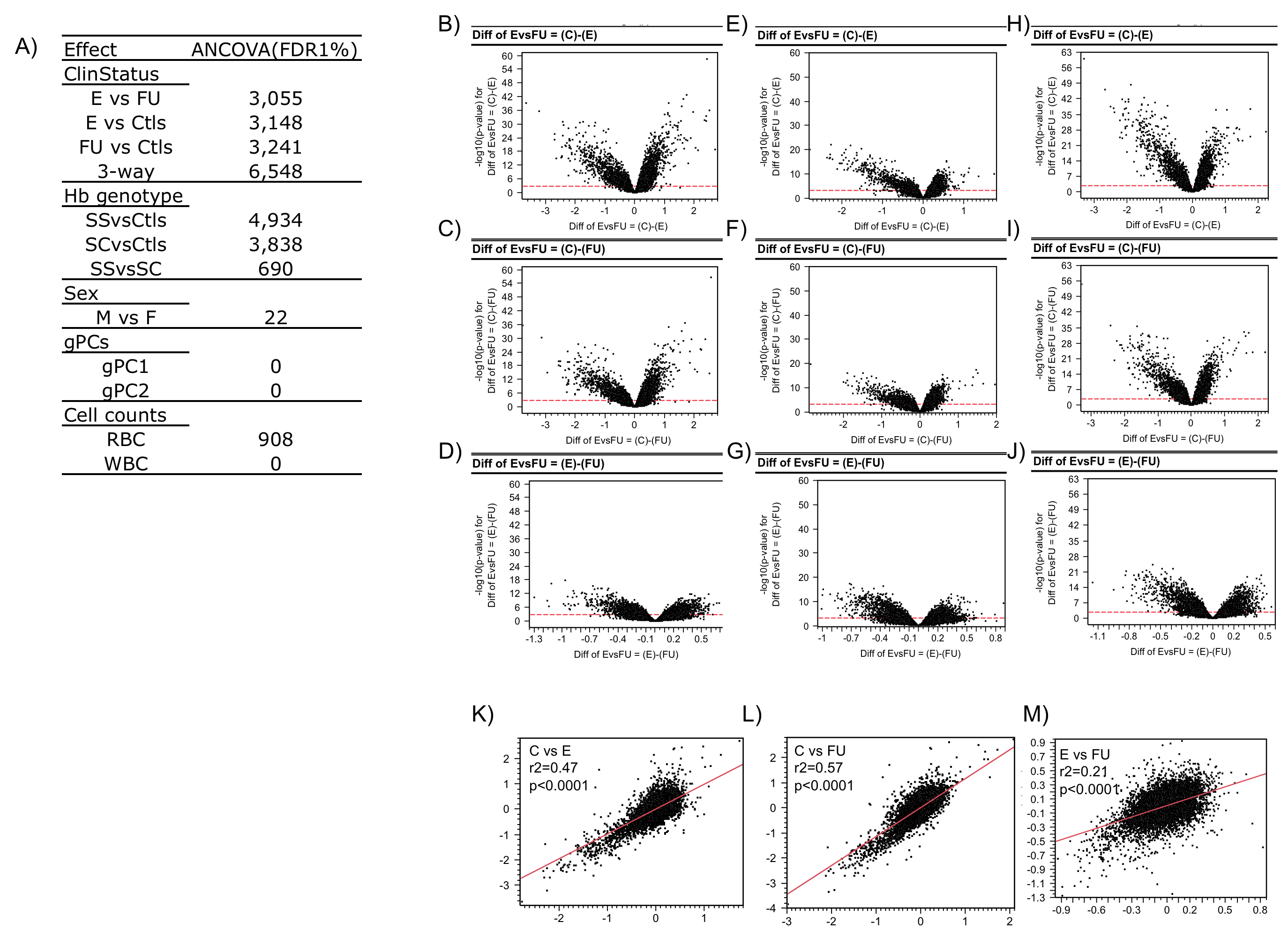

- L)
-
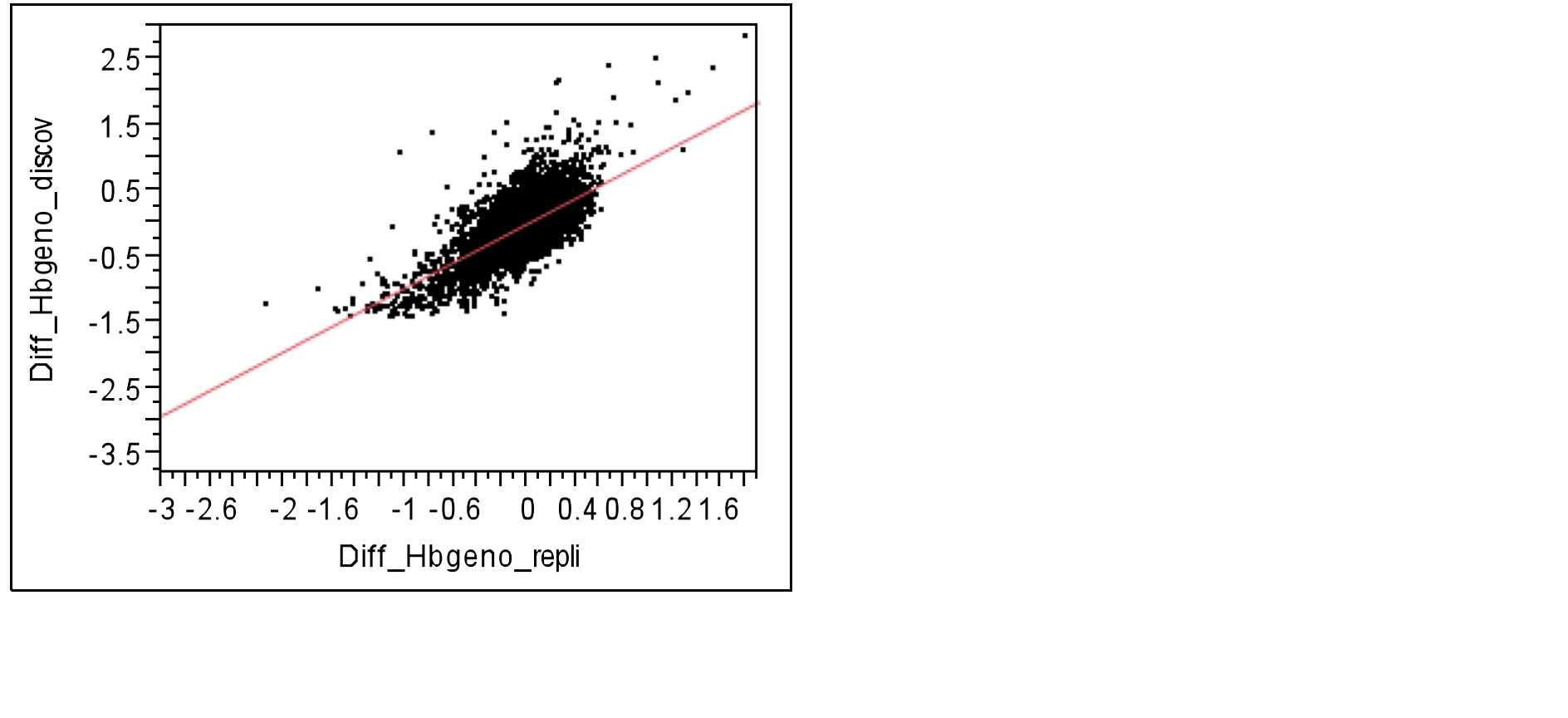

- **Figure S5.**
- **A) Differentially expressed probes in the discovery phase.** Number of probes differentially expressed for the following effects: Clinical Status (E=Entry, Follow-up=FU, and Controls; Ctls), 3-way-contrast (E-vs-FU-vs-Ctls), Hb genotypes (HbSS, HbSC, Ctls), sex (M=males, F= females), and genotypic principal components (gPC1, gPC2). These results were generated using analysis of covariance (ANCOVA, FDR 1%) that accounts for sex, total cell counts (RBC and WBC) and genotypic principal components (gPC1, gPC2).
- **B-J) Volcano plots of differentially expressed probes from ANCOVA (FDR 1%) in discovery, replication, and combined I data sets for the clinical status effect (E vs FU vs Ctls).** Volcano plots showing significance (y-axis, -log10 p-value) vs log2 fold change (x-axis) of all probes analyzed in the contrasts between SCD follow-up and controls using the discovery (B-D), replication (E-G), and combined I dataset (H-J). Probes that are differentially expressed at FDR 1% are above the dotted line colored in red.
- **K-M) Correlation of fold change differences in the discovery and replication phase for the probes that are differentially expressed in the contrasts CvsE (K), CvsFU (L), and EvsFU (M) and HbSSvsC (L)**
- Correlation between fold change differences in the discovery and replication phases for the set of differentially expressed probes between each of the clinical status effects Entry (E), follow-up (FU) and controls (C), or between HbSS and controls (L) (r=0.675). All correlations shown are significant (p<0.001).
- **
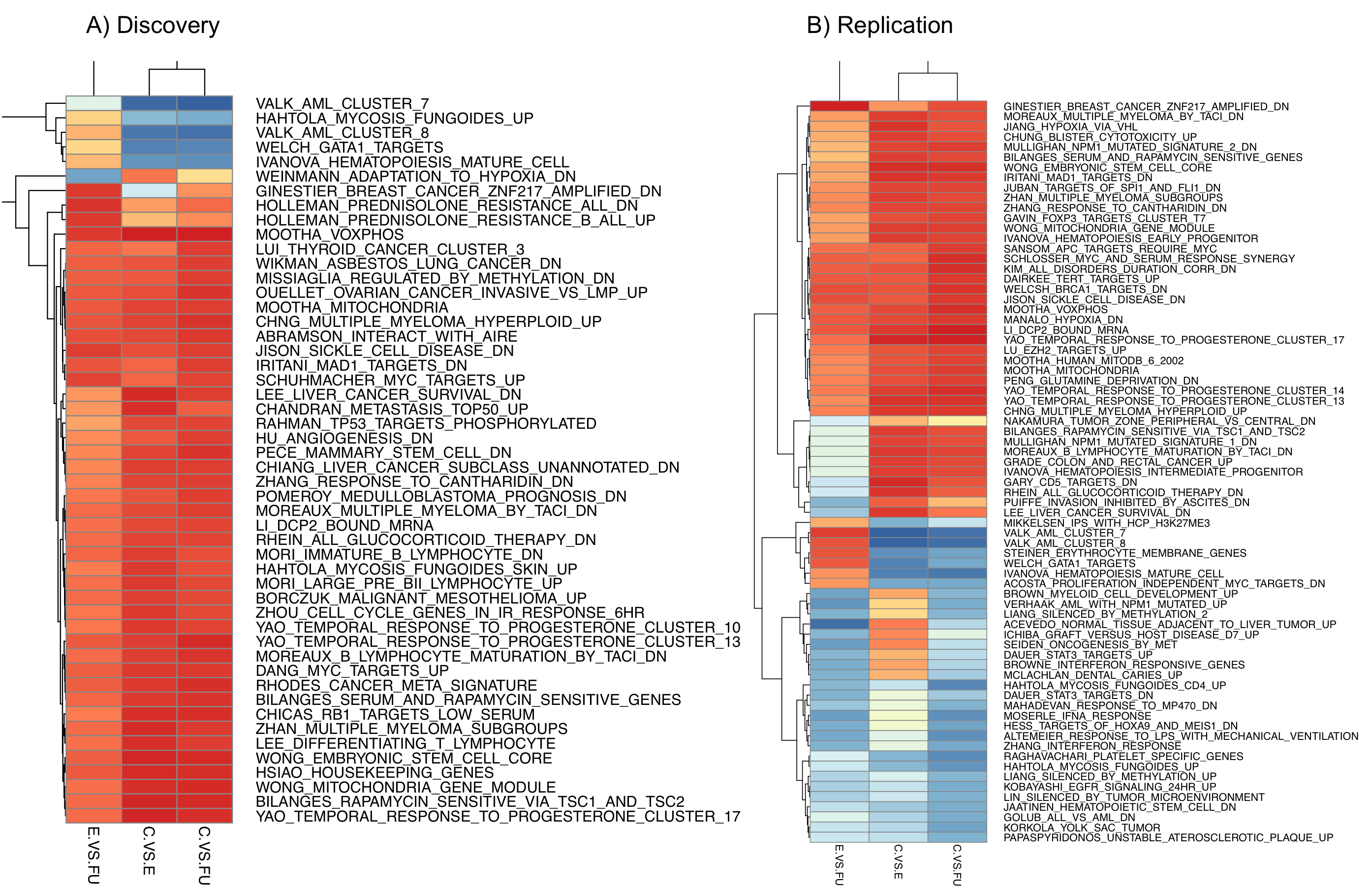
**
- **
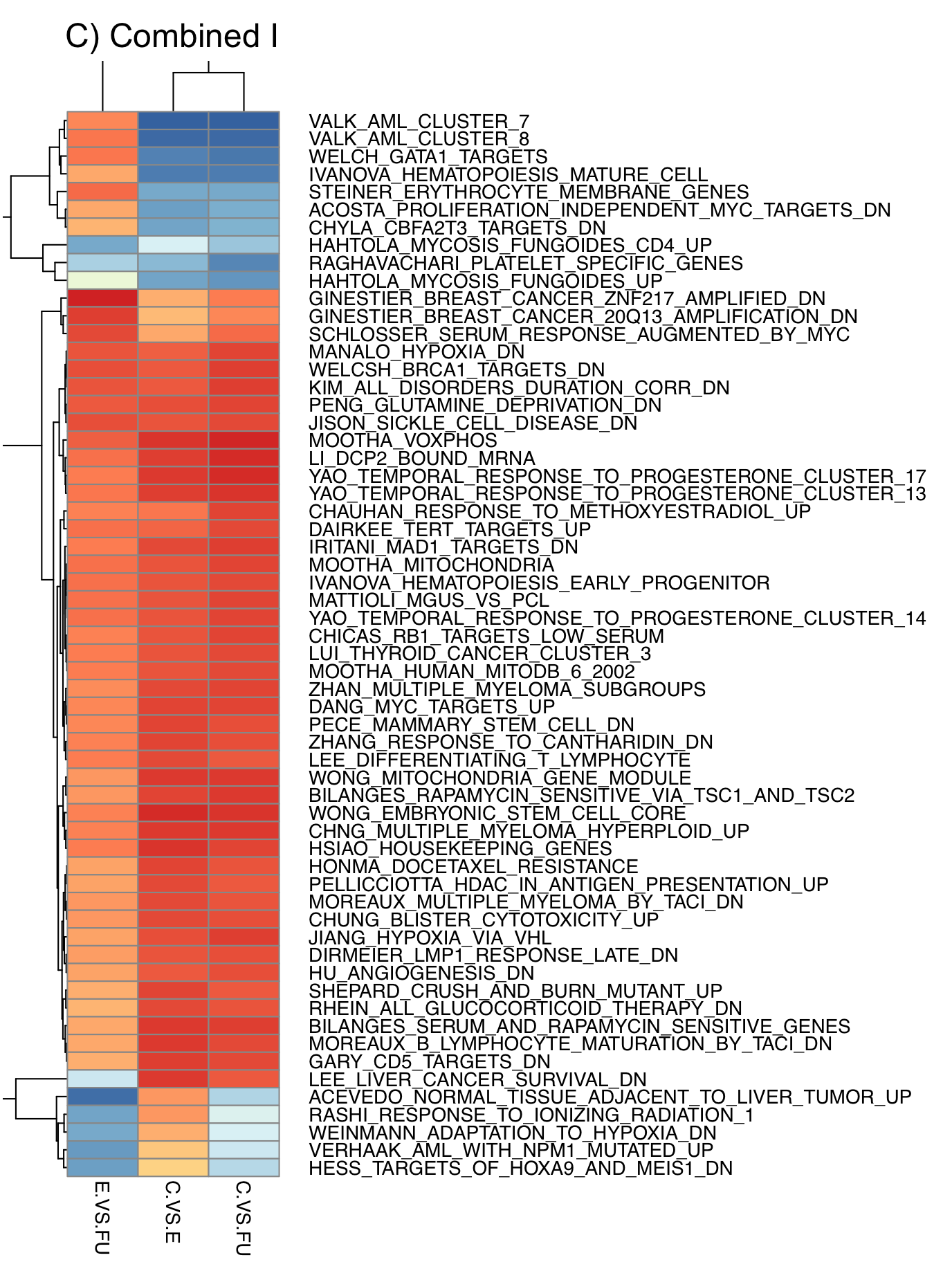
**
- **Figure S6. GSEA**

Gene Set Enrichment Analysis (GSEA) was performed for each contrast of the clinical status effect in the discovery (A), replication (B), and combined I data set (C). This analysis identified biological pathways and sets of individual genes that are significantly enriched in each contrast. Only pathways and modules significantly enriched (FWER P < 0.05) from at least one contrast are shown. Colors in the heat map indicate the enrichment score relative to the contrasted group.

- **
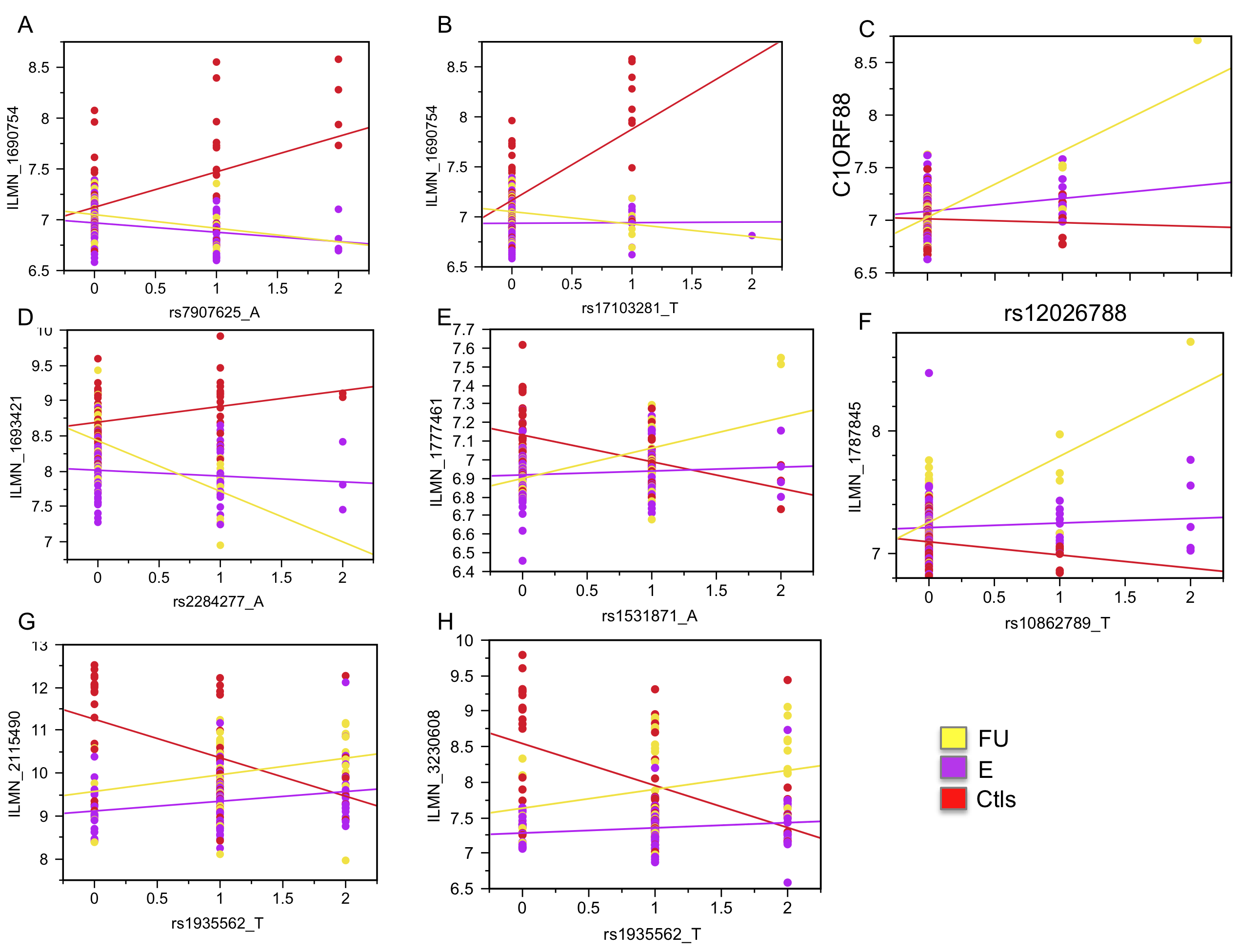
**
- **Figure S7. SNP-by-ClinStatus interaction effects of genes differentially expressed between SCD clinical status.** Using the combined dataset II, a multiple linear regression analysis was performed that accounted for clinical status, sex and cell counts and tested for significant interaction effects for 7002 genes that are differentially expressed between the Clinical Status effect (EvsFUvsCtls). Thirteen peak genome-wide significant interaction effects were identified; eight of which remained significant after running a Q-K mixed model that accounts for relatedness. Five of the 8 interactions are plotted in Fig. 4, and the remainder of interactions are plotted above (A-C are those that remain significant for the Q-K mixed model; D-H the remainder of the 13 interactions). These interaction effects show how the eSNP effect is modulated by clinical status. All interactions are local. The color code for the clinical status is indicated on the right hand side. Expression levels are shown on the y-axis and SNP genotypic class on the x-axis.


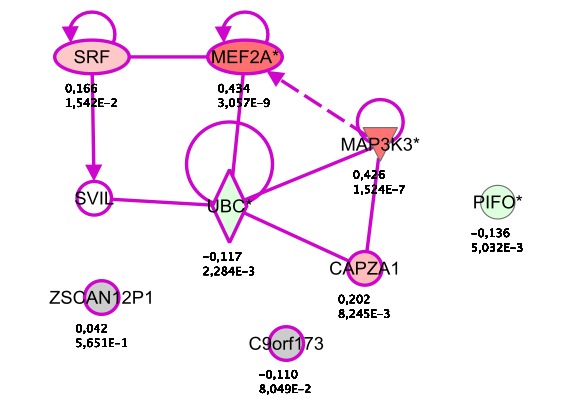


**Figure S8. Interaction network** of the 6eSNP genes. Three of these eSNP genes were part of the network : CAPZA1, SVIL, and MEF2A hinting to their shared biological mechanisms in SCD. Under each node is the gene’s corresponding log2 fold change and adjusted p-value for differential expression between C and E. Each node is colored based on these values. The network was generated through the use of IPA (Ingenuity Systems, [www.ingenuity.com](http://www.ingenuity.com/)).


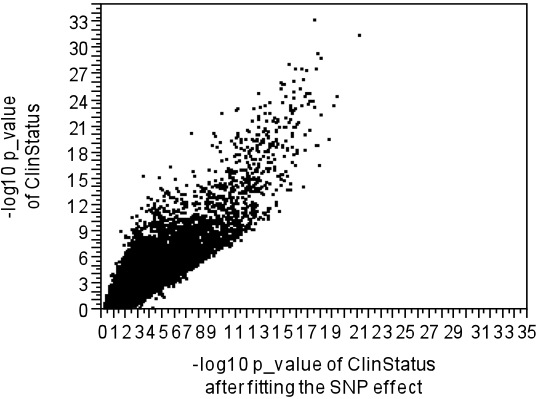


**Figure S9. Correlation of –log10 p-values of Clinical Status effect** **before and after fitting the SNP effect.** A full ANCOVA model with and without the SNP effect was run using Comined dataset II for all expressed genes. Bonferonni corrections were applied. The p-values for ClinStatus for all the genes were extracted and contrasted for the two models. A relatively high degree of correlation is shown (R^2^= 0.788) suggesting that quantitatively most of the transcriptional signal differentiating the clinical categories is robust.

**Supplementary Table**

A

-
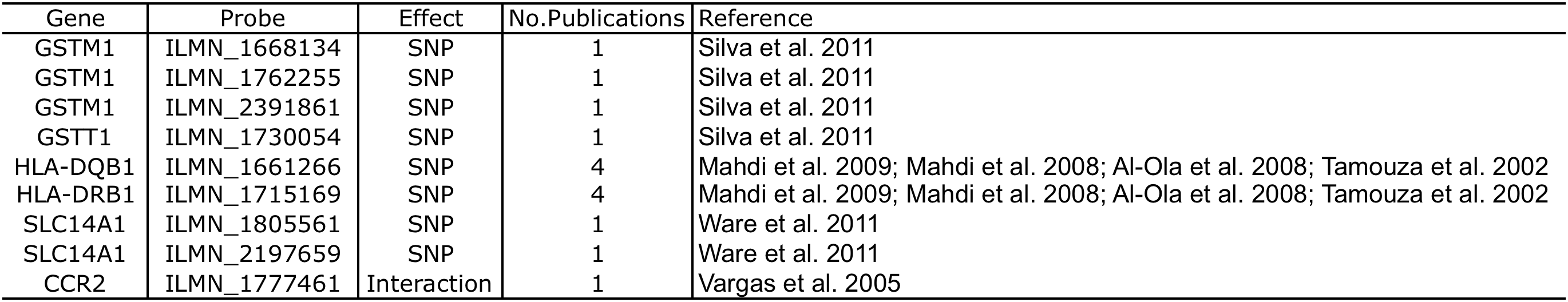

- B
-
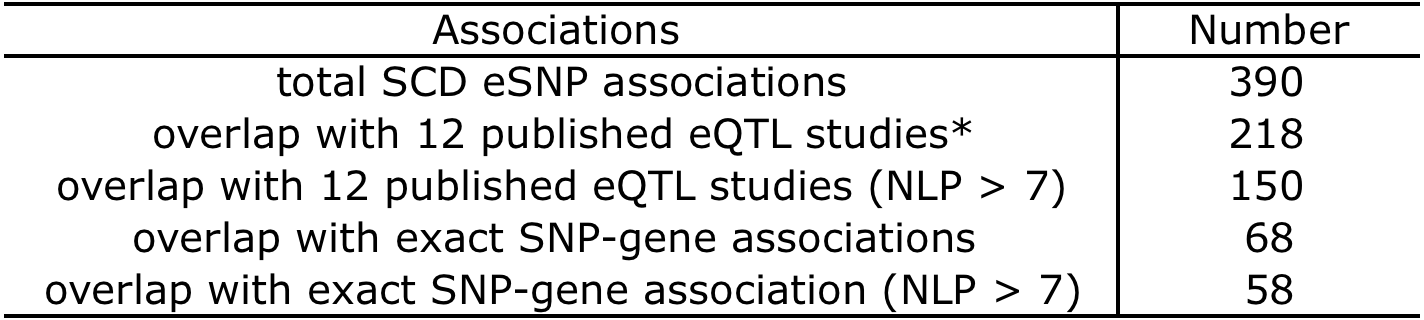

- * References: Dimas09_Tcell, Dimas09_Fibro, Dimas09_Lympho, Montgomery10_exon, Montgomery10_transcript, Myers, Pickrell10_eQTL, Pickrell10_sQTL, Schadt, Stranger, Vayrieras_Pval, Zellers10
- **Table S1.**
- **A) 5 eSNP genes previously associated with SCD through candidate gene studies.** The list of candidate genes associated with SCD was obtained from the PhenoPedia database (http://www.hugenavigator.net/HuGENavigator/startPagePhenoPedia.do). Genes with genetic markers that had previously been associated with SCD were overlapped with genes under eSNP control from the combined dataset. The eight gene and their respective references to previous reports on SCD are shown in the table above.

**B) Overlap between genes associated with eSNPs in the present study and previous eQTL studies available through the eQTL database** (**http://eqtl.uchicago.edu/cgi-bin/gbrowse/eqtl/**). The table shows the number of genes in our study (detected at genome-wide significance) that overlap with genes reported in the twelve published studies indicated. The comparison includes all genes reported in these studies, as well as those that are significant at NLP > 10-7. The total number of genes that overlapped is two hundred eighteen, one hundred and fifty of which are significant. Sixty eight are exact gene-SNP pairs, with fifty eight being significant.

**Supplementary File 1.** Microsoft Excel Spreadsheet of ANCOVA results for differentially expressed genes between EvsFUvsCtls in the discovery, replication, combined I and II data sets.

**Supplementary File 2.** Microsoft Excel Spreadsheet of ANCOVA results for differentially expressed genes between Hb genotypes in the discovery, replication, and combined data sets.

**Supplementary File 3.** Microsoft Excel Spreadsheet of the participants and a description of experimental variables in discovery, replication, and combined data sets.

**Supplementary File 4**. Microsoft Excel Spreadsheet of 390 eSNP associations for model 1 in the combined II data set.

**Supplementary File 5.** Spreadsheet containing the GSEA results for both the discovery (discov) and replication (rep) cohorts. Functional categories and their differential expression trend (Up or dow-regulatation) for each contrast (Entry (E) vs Control (C), Follow-Up (FU) vs Control (C) and Follow-Up (FU) vs Entry (E)) as well as level of significance and the expression fold change are included. NA = not significant.

- **References**
- **1. Hanchard N, Elzein A, Trafford C, Rockett K, Pinder M, et al. (2007) Classical sickle beta-globin haplotypes exhibit a high degree of long-range haplotype similarity in African and Afro-Caribbean populations. BMC Genet 8: 52.**
